# Panic disorder aging characteristics: The role of telomerase reverse transcriptase gene and brain function

**DOI:** 10.3389/fnagi.2022.835963

**Published:** 2022-08-05

**Authors:** Huachen Ding, Yuan Zhong, Na Liu, Huiqin Wu, Huazhen Xu, Yun Wu, Gang Liu, Shiting Yuan, Qigang Zhou, Chun Wang

**Affiliations:** ^1^Nanjing Brain Hospital Affiliated to Nanjing Medical University, Nanjing, China; ^2^Functional Brain Imaging Institute of Nanjing Medical University, Nanjing, China; ^3^School of Psychology, Nanjing Normal University, Nanjing, China; ^4^Cognitive Behavioral Therapy Institute of Nanjing Medical University, Nanjing, China; ^5^Department of Clinical Pharmacology, School of Pharmacy, Nanjing Medical University, Nanjing, China

**Keywords:** telomerase reverse transcriptase gene, DNA methylation, aging, panic disorder, regional homogeneity

## Abstract

Panic disorder (PD) causes serious functional damage and disability and accelerates the process of individual aging. The pathological basis of PD is the same as that of age-related diseases, which is proposed as a new viewpoint in recent years. Memory decline and social functional impairment are common manifestations of accelerated aging in PD. The function of telomerase reverse transcriptase (TERT) and telomere length (TL) is abnormal in patients with aging and PD. However, the molecular mechanism behind remains unclear. The purpose of this study was to explore the relationship between TERT gene expression (including DNA methylation) and the changes in PD aging characteristics (memory and social function). By TERT gene knockout mice, we found that loss of TERT attenuated the acquisition of recent fear memory during contextual fear conditioning. This study reported that a significantly lower methylation level of human TERT (hTERT) gene was detected in PD patients compared with healthy control and particularly decreased CpG methylation in the promoter region of hTERT was associated with the clinical characteristics in PD. Regional homogeneity (ReHo) analysis showed that the methylation of hTERT (cg1295648) influenced social function of PD patients through moderating the function of the left postcentral gyrus (PCG). This indicates that the hTERT gene may play an important role in the pathological basis of PD aging and may become a biological marker for evaluating PD aging. These findings provide multidimensional evidence for the underlying genetic and pathological mechanisms of PD.

## Introduction

Panic disorder (PD) impairs social function and memory in patients and is at risk for accelerated aging (Perna et al., 2016). Memory decline and social functional impairment are common manifestations of accelerated aging in PD (Markowitz et al., 1989; Yochim et al., 2013). It is commonly co-morbid with age-related diseases including depression (Kessler et al., 2006) and Alzheimer’s disease (Verhoeven et al., 2015). PD and other age-related diseases may have a common pathophysiological basis, which is proposed as a new viewpoint in recent years. The genetic mechanism in PD aging is unclear. Telomere length (TL) is closely related to human aging, especially in memory decline and social function impairment (Verhoeven et al., 2015). The expression of the telomerase reverse transcriptase (TERT) gene plays an important role in regulating TL. The lower expression of the TERT gene generally causes the shorter TL and the hypomethylation or demethylation of the human TERT (hTERT) gene usually reduces hTERT gene expression (Guilleret and Benhattar, 2003). Telomere erosion-mediated cellular senescence is widely used in the study of mental disorders (Lindqvist et al., 2015). Shorter TL has been reported to be relevant to the severity of PD. TERT gene mutation and TERT-knockout can cause stem cell dysfunction, leading to premature aging (Perna et al., 2016). The hTERT gene expression plays an important role in neuronal development (Klapper et al., 2001) and emotional processing, particularly fear processing, fear regulation, and anxiety (Meyer-Lindenberg and Weinberger, 2006). The associations between hTERT gene, depression, and age-related diseases has been confirmed by several studies (Gonzalez-Giraldo et al., 2016; Michalek et al., 2017). The correlations between TERT gene function and anxiety syndrome have been reported in both humans and animals (Teyssier et al., 2012; Kim et al., 2017). Overexpression of the TERT gene is associated with reduced anxiety-like behaviors in male mice, affecting male mice’s function in terms of social preference and social competence (Kim et al., 2017). The positive correlation between hTERT gene expression and anxiety level is reported in depressed patients (Teyssier et al., 2012). These findings suggest that the expression of hTERT gene may be involved in the pathology of PD aging.

DNA methylation has been shown to crucially modify gene expression and is an epigenetic process involved in development and aging and sensitive to environmental influences. The differential contribution of DNA methylation in those at risk for PD and has been supported by twins (Alisch et al., 2017) and epidemiologic studies (Bartlett et al., 2017). The hTERT methylation is mostly studied in cancer research (de Wilde et al., 2010) but one study reported the hTERT methylation frequency in Alzheimer’s disease (AD) differed from elderly controls (Silva et al., 2008). Since the genetic effects of the disease may occur by affecting the brain, moreover changes in brain function are more pronounced than changes in brain structure in patients with PD, the investigation of brain function changes associated with the genetic risk for panic disorder is one of an important strategy to find the hTERT gene methylation role. To date, such investigations have mainly focused on the associations of some risk genes with neuroimaging measures or a probed for an “imaging intermediate phenotype.” In PD, the study of risk genes is involved in many neurotransmitters and extends to telomerase and other systems gradually (Iurato et al., 2017; Emeny et al., 2018). The amygdala and hippocampus have been commonly reported as neural intermediate phenotypes related to the functional catechol-O-methyltransferase (COMT) gene and so on (Inoue et al., 2015; Sobanski and Wagner, 2017). However, the imaging intermediate phenotype associated with hTERT gene methylation is unclear in PD. Growing evidence suggests that TL partially predicts variations in the brain and aging (Ain et al., 2018; Powell et al., 2019) and the associations between TL and brain function of the amygdala, hippocampus, cuneus, mPFC, and PCG are reported (King et al., 2014; Kim et al., 2016; Powell et al., 2019; Zeng et al., 2019). The effect of TERT gene expression on brain function has been reported in animals (Lee et al., 2010). However, the imaging intermediate phenotype associated with hTERT gene methylation is unclear in PD.

Based on the above-mentioned evidence, we hypothesized that hTERT methylation: modulated brain functional alterations, may influence the amygdala/hippocampus/postcentral gyrus (shared alterations in regions implicated in aging) to increase the possibility of PD aging. The purpose of this study was to explore the relationship between TERT gene expression (including DNA methylation) and the changes in PD aging characteristics (memory and social function). Further, enrich the genetic imaging mechanism of PD aging. To provide biological basis for early prevention, early detection and early intervention to delay the aging process of PD patients.

## Materials and methods

### Study approval and human subjects

**FIGURE 1:**
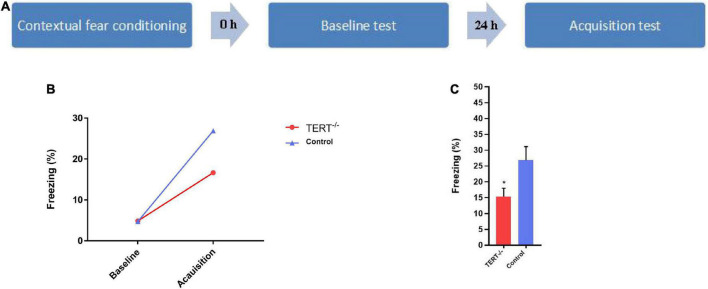
The impairment of recent fear memory in TERT^–/–^ mice in contextual fear conditioning. **(A)** Schematic representation of the behavioral protocol. **(B)** There was no difference in the probability of freezing behavior in the two groups at baseline during the test, but both groups have a significant increase of freezing time percentage after 24 h. **(C)** The acquisition of fear memory has a significant difference between TERT^–/–^ mice and normal TERT gene mice (*P < 0.05), the acquisition of recent fear memory in TERT^–/–^ mice is lower than this in control group.

The original sample consisted of 32 eligible PD patients and 22 eligible Healthy controls. They were recruited from Nanjing brain hospital affiliated with Nanjing Medical University through outpatient and public advertising. The selection criteria for PD subjects were as follows: (1)18–55 years old. (2) Right handedness. (3) Ability to cooperate with all tests and complete them. (4) No other illnesses, no psychotherapy or medical treatment in the past 6 months. We used mice for a contextual recent fear memory acquisition test (Figure 1A). Four trained psychiatrists used the Mini-international Neuropsychiatric Interview (MINI) to screen all PD patients according to DSM-IV criteria. The scores of the age-match healthy controls on the Hamilton Anxiety Rating Scales (HAMA) should < 7.

The study was approved by the Ethics Committee of the Nanjing Brain Hospital, affiliates of Nanjing Medical University. All subjects obtained informed consent. The HAMA scale was used to assess the level of anxiety in all subjects. The Panic Disorder Severity Scale (PDSS) was used to assess the severity of panic symptoms in PD patients.

### Animals

All animal procedures were approved by the Institutional Animal Care and Use Committee of Nanjing Medical University. TERT^–/–^mice were backcrossed to FVB/N mice to produce mice heterozygous for TERT. The first generation of TERT ± mice (F1) was used for the production of TERT^–/–^ mice (F2). All experimental subjects were from the fourth or fifth generation after hybridization of TERT^–/–^ mice (F2). Two to three-month-old male mice were used for behavioral tests. In total, there were 15 TERT^–/–^ mice and 21 normal TERT gene mice for testing. Compared with WT mice, the telomerase catalytic activity was not detected in the hippocampus of TERT^–/–^ mice.

### DNA methylation data

Peripheral venous blood was drawn from patients by certified nurses at Nanjing Brain Hospital, affiliates of Nanjing Medical University. DNA was extracted from whole peripheral blood (stored in EDTA tubes) with standardized salting out methods and quantified by NanoDrop 2000 (NanoDrop technologies, Wilmington, DE, United States). Genomic DNA (1 μg; concentration, 20 ng/μL) was bisulfite converted using the Zymo EZ DNA-methylation kit (ZYMO, CA, United States). The multiple-PCR was performed to amplify the bisulfite-modified DNA sequence using indexed primers. The detection of hTERT methylation was performed on Illumina Hiseq (Illumina, CA, United States) using bidirectional sequencing verification with 2 × 150 bp sequencing mode according to the manufacturer’s protocol and to analyze the results. The methylation levels of the hTERT gene promoter were analyzed by MethylTarget™ (Genesky Biotechnologies Inc., Shanghai, China). CpG islands located in the promoter of the hTERT gene were selected from 2 k upstream of transcriptional start site (TSS) to 1 k downstream of the first exon according to the following criteria: (1) 200 bp minimum length; (2) the ratio of observed/expected dinucleotides CpG > 0.60; (3) the content of GC should be no less than 50%. Finally, we selected 7 CpG regions of the hTERT gene promoter including 197 CpG sites (Supplementary Table 1).

### Regional homogeneity analyses

The ReHo map of each subject was produced by calculating the Kendall consistency coefficient (KCC) of the ranked time series of a given voxel and its nearest 26 neighboring voxels. The intracranial voxels were extracted to make a whole-brain mask without the non-brain tissue. For standardization purposes, each ReHo map was divided by its own global mean KCC within the whole-brain mask. The generated ReHo maps were spatially smoothed with a 4 × mm 4 mm × 4 mm FWHM Gaussian kernel.

### Behavioral tests

We used mice for a contextual recent fear memory acquisition test. Mice were habituated in a conditioning chamber (18 cm × 18 cm × 30 cm) which had a grid floor that could be energized and connected to a shock generator. Habituation lasted for 3 min, and percentage of freezing time during those 3 min served as the baseline (freezing was defined as no movement detected for 2 s). After habituation, a foot shock (unconditioned stimulation, 2 s, 0.75 mA) was delivered to the mice. After the shocks, mice remained in the chamber for 1 min and were then returned to their cage. 24 h later, the mice were put back in the chamber, and given the same foot shock for 3 min to obtain the fear acquisition value. During the training session, the mice’s behavior was captured by a CCD-camera and the freezing behavior was analyzed by the freezing behavior analyzing software (FreezeScan version 2.00, Clever Sys Inc.).

### Statistical analysis

Data analysis was carried out using SPSS software. Differences in dimensional sample characteristics were tested through independent samples *t*-tests; a Two-sample *T*-test was used to compare methylation levels of the hTERT gene between PD patients and healthy controls. Differences in categorical variables were tested using Chi-square tests. Spearman correlations were adopted for bivariate correlation analyses. All data met the normality assumption. We used the GraphPad Prism version 7 to draw the graphs.

To examine the effects of ReHo and hTERT promoter methylation in PD, multiple regression analysis in SPM12 was performed with PD symptoms, methylation, and imaging data. Then, we used REST 1.8 to find the overlap of regions in relation to methylation and PDSS scores. A linear regression model was performed to discuss the interaction effects, corrected for age and gender.

## Results

### The deletion of telomerase reverse transcriptase gene attenuated the acquisition of recent fear memory during contextual fear conditioning

Figure 1B shows that there was no difference between TERT^–/–^ mice and wildtype mice in the baseline test. Twenty-four hours after the baseline test, mice were exposed to the same fear chamber to evoke fear memory, and the percentage of freezing time was significantly higher than in the first baseline period for both TERT^–/–^ and wildtype mice. However, the fear acquisition was significantly inhibited in TERT^–/–^ mice compared with wild-type mice (Figure 1C).

### Demographic and clinical characteristics

The demographic and clinical features of the 32 PD patients and 22 healthy controls at the first stage are listed in Table 1. There were no statistically significant differences in age (*t* = 0.73, *p* = 0.94), gender (χ^2^ = 0.11, *p* = 0.74), or education level (*t* = 1.7, *p* = 0.09) between PD patients and healthy controls. There were significant differences in HAMA-14 scores (*t* = –11.8, *p* = 0.00) between PD and HC.

**TABLE 1:** Demographic and clinical characteristics of total samples.

	PD (SD)	HC (SD)	Different	
Subjects (*n*)	32	22		
Male/Female	16/16	12/10	0.11^a^	0.74
Age (year)	33.1 (7.4)	33.3 (7.2)	0.73^b^	0.94
HAMA-14	20.3 (7.0)	2.2 (1.9)	–11.8^b^	0.00
Education (years)	13.9 (3.3)	15.6 (3.8)	1.7^b^	0.09

a = χ^2^; b = independent sample *t*-test. PD, panic disorder; HC, healthy control; SD, standard deviation; HAMA-14, the total scores of Hamilton Anxiety Scale 14 Items.

### Lower methylation in the promoter of human telomerase reverse transcriptase gene in panic disorder associated with high severity of panic disorder

**FIGURE 2:**
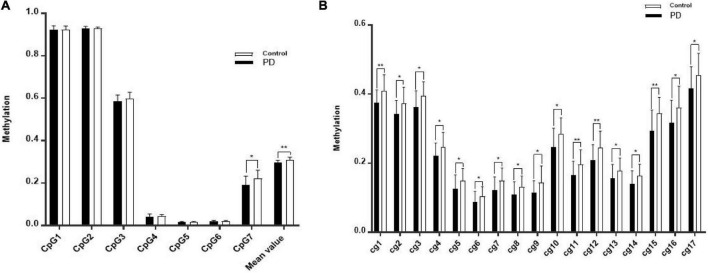
**(A)** The methylation of CpG islands in *hTERT* gene in the discovery sample of patients with PD and HCs. **(B)** The lower methylation in PD compared with controls. “*” Significant at *P* < 0.05; “**” significant at *P* < 0.01.

The methylation of hTERT gene differed significantly between PD patients and healthy controls (Figure 2), with PD patients exhibiting decreased average methylation (*p* = 0.0071). The CpG7 Island of the hTERT gene promoter was hypomethylated in PD patients compared to healthy controls (*p* = 0.0133). There were 23 CpG sites in CpG7 region and of them, 13 (cg5-17) differential CpG sites were significantly hypomethylated. In addition, hypomethylation was detected in 4 CpG sites in other islands in the hTERT gene promoter in PD patients. These 17 CpG sites were analysis for correlations with clinical characteristics.

**FIGURE 3:**
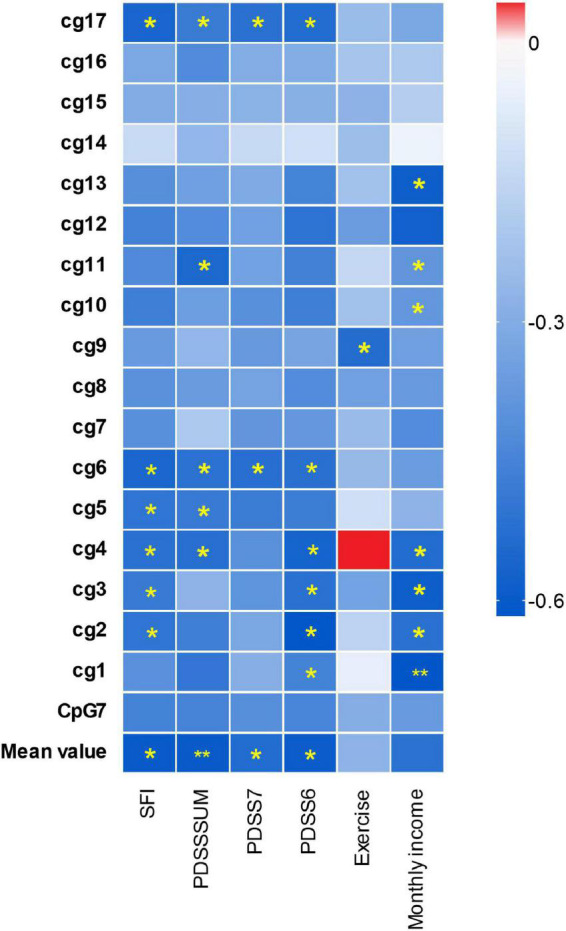
Differential hTERT methylation and the clinical characteristics in panic disorder. The *x*-axis shows the clinical characteristics of PD patients; the *y*-axis shows the hTERT genes average methylation, the different methylation of the gene CpG islands and cg sites. The color of the box shows the Pearson correlation coefficient (red for positive correlation, blue for negative correlation). The color intensity represents the degree of enrichment. “*” indicates enrichment is statistically significant (*p* < 0.05), “**” indicates *p* < 0.01.

The significant results are shown in Figure 2, located inside CpG7, the methylation level of 3 CpG sites located inside CpG7 was significantly negatively associated with monthly income and 1 CpG site was significantly positively associated with the level of realistic physical exercise (Figure 3). No significant correlations were found between methylation level and gender, course of the disease, age, the age of onset.

The Pearson correlation analysis showed the negative relationship between the methylation alterations of the hTERT gene and the severity of PD, include total PDSS, PDSS6, PDSS7, and SFI scores (social function impairment scales) (Figure 3). There were significant inverse relations for the methylation mean and the scores of all four PDSS scales. The methylation level of 2 CpG sites (cg6 and cg17) located in the CpG7 region were negatively relevant to the scores of all the PDSS scales.

### Hypomethylation of the human telomerase reverse transcriptase gene promoter and regional homogeneity analysis

**FIGURE 4:**
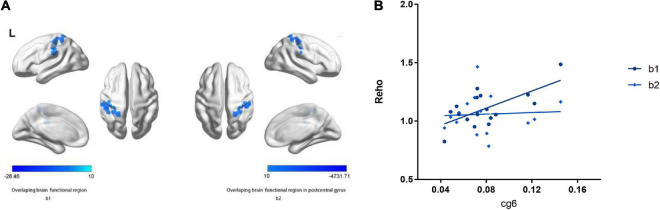
Main effects of the hTERT gene promoter methylation on ReHo. **(A)** b1 shows decreased ReHo in left inferior parietal lobule, postcentral gyrus negative associated with cg1295648 methylation level and social function scores in PD patients. b2 shows decreased ReHo in left postcentral gyrus negative related to cg1295648 methylation level and social function scores in PD patients. **(B)** The methylation of cg1295648 correlates with the value of ReHo in b1, b2 positively.

In the PD group, there were positive correlations between ReHo and methylation levels at hTERT average (Table 2 and Figure 4).

**TABLE 2:** Significant clusters show the effects of PD and hTERT methylation level on regional homogeneity.

	MNI coordinates			Cluster voxels	*T*-value	Area	Hemisphere
	*x*	*y*	*z*				
hTERT average	–42	–48	39	133	9.4383	PCG	L
				129		IPL	L
cg6	–54	–24	33	191	5.8263	PCG	L
				109		IPL	L
SFI	–54	–24	27	114	–8.2444	PCG	L
				87		IPL	L
	–54	–24	15	67	–6.4218	Thalamus	L

hTERT ave: mean value of hTERT promoter methylation. SFI: significant clusters on ReHo association with the social function impairment of PD patients.

### Correlations between social function impairment, panic symptom severity and human telomerase reverse transcriptase-modulated differences in regional homogeneity

No direct association between ReHo and the total PDSS score was found, however, there was a negative correlation between SFI score and ReHo in the left PCG and IPL. The aberrant ReHo in left PCG and IPL was linked to both hTERT methylation and SFI in PD patients. Average ReHo values were hence extracted from the overlapping region of the two clusters showing significant effects of hTERT methylation and SFI in PD group; 2 significant overlapping clusters, b/b1, were found (Table 3 and Figure 4). Within these clusters, negative correlations between social function damage and hTERT gene average/cg6 (cg1295648) methylation were observed (hTERT average: *r* = –0.601, *p* = 0.011; cg6: *r* = –0.557, *p* = 0.02). The results also showed a significant negative correlation between ReHo of b1/b and hTERT gene average/cg6 (cg1295648) methylation (hTERT average: *r* = –0.757, *p* < 0.0001; cg6: *r* = –0.736, *p* = 0.001). Because the mean value of hTERT methylation could not be accurately quantified, the cg6 was chosen as an independent variable to study the interaction between brain function and methylation. To better understand the role of PCG and IPL, the values of the b1 cluster were divided into the values of b2 (left PCG)/b3 (left IPL) clusters (Figure 4). ReHo in left PCG (b2) and IPL (b3) was related to severity of PD (PCG: *r* = –0.609, *p* = 0.009; IPL: *r* = –0.569, *p* = 0.017).

**TABLE 3:** Significant overlapping clusters that associated with hTERT gene methylation and the degree of social functional impairment in PD.

	MNI coordinates			Cluster voxels	Region
Methylation	*x*	*y*	*z*		
hTERT ave(b)	–45	–33	45	65	Postcentral_L
				49	Parietal_Inf_L
cg6(b1)	–54	–24	30	67	Postcentral_L
				46	Parietal_Inf_L
cg6(b2)	–54	–24	39	56	Postcentral_L

hTERT ave: mean value of hTERT promoter methylation. b1: the significant overlapping clusters that associated with the methylation of cg1295648 and the degree of harm of patients’ social function. b2: the significant overlapping clusters located left postcentral gyrus, that associated with the methylation of cg1295648 and the degree of harm of patients’ social function.

After controlling for age and sex, the linear regression models revealed an interaction between hypomethylation of cg6 and ReHo modulated by the CpG site in the left PCG (b2) (*p* = 0.043, Table 4). In PD, the hypomethylation of the CpG site decreased social function by moderating the function of left PCG. The moderating effect equation is as follows: *Y* = 132.715 × 1 X–8.478 × 1 X (*R*^2^ = 0.634, X1 = cg6 methylation level, *X* = the value of b2 clusters) (Figures 4A,B).

**TABLE 4:** Linear regression models of brain function and interaction effect with cg1295648 CpG methylation in social function damage in PD.

		B	*t*	*P*	*R* ^2^	*R*^2^ change	Sig. F change
Model 1	cg6(a)	–8.968	–0.517	0.613	0.551	0.551	0.004**
	overlaping region(b1)	–8.571	–2.737	0.016*			
Model 2	A	–23.124	–1.369	0.194	0.666	0.115	0.054
	b1	–9.301	–3.295	0.006*			
	c1	123.272	2.117	0.054			
Model 3	a	–13.614	–0.746	0.468	0.493	0.493	0.009**
	PCG_L_overlap(b2)	–7.068	–2.241	0.042*			
Model 4	A	–26.98	–1.572	0.14	0.634	0.141	0.043*
	b2	–8.478	–2.973	0.011*			
	c2	132.715	2.239	0.043*			
Model 5	a	–14.406	–0.862	0.403	0.531		0.005**
	IPL_L_overlap(b3)	–6.939	–2.563	0.023*			
Model 6	a	–21.95	–1.144	0.273	0.554		0.422
	b3	–6.255	–2.187	0.048*			
	c3	53.131	0.829	0.422			

Linear regression models for the association of hTERT promoter methylation-modulated alterations in ReHo (b1, b2, b3), social function damage and M-value cg1295648 methylation are shown for a model adjusted for sex, age, monthly income. Model 2/4/6 included interaction terms (c1/c2/c3) for cg1295648 CpG methylation and mention value of ReHo in b1/b2 located in left postcentral gyrus/b3 brain region located in left inferior parietal lobule. *p < 0.05, **p < 0.01.

## Discussion

The ability of functional brain imaging techniques to explore genes is well-recognized. Recent PD studies have used functional imaging to examine the effect of a panic disorder-related brain-based intermediate phenotype on neural activity (Gechter et al., 2019; Gottschalk et al., 2019). But few studies have shown the neural intermediate phenotype of anxiety in relation to methylation (Oh et al., 2013; Ziegler et al., 2015). This study is the first to demonstrate that ReHo in specific brain regions is affected by hTERT promoter methylation (especially, cg1295648) in PD. This study reported significant hypomethylation of the hTERT promoter in panic disorder patients as well as significantly lower methylation of CpG sites in the CpG7 promoter region. The methylation level showed significant correlations with disease symptoms (severity of PD, especially the degree of social function damage), sociological data related to stress (e.g., per-capita income) (Powell-Wiley et al., 2020) and the engagement of protective behaviors (e.g., exercise) (Nomikos et al., 2018). The association between higher frequency of exercise/higher income and increased methylation is consistent with other studies that suggested exercise can delay telomere shortening and lower-income can accelerate telomere shortening (Nomikos et al., 2018; Powell-Wiley et al., 2020). These results are corroborated by previous findings that TL may be shorter in PD patients than in healthy controls (Lindqvist et al., 2015).

Genes affect the changes in brain structure and function, which are thought to be mediated by epigenetics. DNA methylation is a widely studied epigenetic modification. 60 reliable studies involving the relationship between DNA methylation and human brain structure/function are reviewed (Wheater et al., 2020). These studies report DNA methylation-MRI associations for psychiatric disorders, including schizophrenia, major depression, post-traumatic stress disorder, social phobia, and so on. All studies used alternative tissues to detect methylation, and 46 of them explored the relationship between DNA methylation and brain structure and function by collecting blood samples. Combining genetic and fMRI data, this study observed that the TERT gene may play an important role in memory and functional impairment associated with brain aging and is involved in the pathological mechanisms of PD. It has been inferred that the gene-brain-behavior relationship can be largely attributed to the TERT gene, and its methylation alterations may lead to symptoms (e.g., memory and functional impairment) related to aging. This is the first study to demonstrate that decreased ReHo in left PCG and IPL was associated with the TERT gene in patients with PD. At the same time, social function impairment scores, represented by the PDSS scale, displayed a negative association with ReHo in left PCG and IPL in PD patients. The SFI of PD patients has a relationship with decreased ReHo in left PCG which is regulated by the hTERT gene methylation. Previous studies have shown that neurobiological alterations of inferior parietal lobule (Magnotta et al., 2014; Lai and Wu, 2016) and postcentral gyrus (Maggioni et al., 2019) are implicated in the pathophysiology of PD, and some of the negative alterations they reported were consistent with our research (Lai and Wu, 2016). Considering the IPL is important for the mental representation of social space (Husain and Nachev, 2007), the IPL abnormality in PD makes it difficult for people to respond to societal stimulation and is negatively related to the severity of social avoidance (Irle et al., 2014; Cardillo et al., 2018). The PCG has also been linked to social avoidance, with two studies reporting that PCG abnormalities were related to stronger social avoidance (Syal et al., 2012). Decreased activity of the PCG in response to sad faces has been observed in people with high attachment avoidance (Li et al., 2014). In healthy people, the PCG and the IPL are thought to play putative roles in avoidance behaviors and responses to the immediate intention of other persons (Conty et al., 2012). It means these functional changes in IPL and PCG may play a key role in social function related to PD, and the moderating model in our study supports this view. Overexpression of TERT was associated with reduced social interaction and decreased preference for novel social interaction (Rhee et al., 2018). Telomere shortening has been observed in autistic children with social interaction disorders (Li et al., 2014). TL in the parietal cortex has a potential association with its brain function (King et al., 2014; Cardillo et al., 2018). These studies built on the findings of the studies discussed above to suggest that the changes in TERT expression could indirectly affect the social function of individuals with PD. These hTERT promoter methylation-modulated brain functional alterations in ReHo were related to SFI in PD patients. PCG is the main region affected by hTERT gene methylation and has a major impact on social functioning in the PD group, possibly owing to the unique role of PCG in abnormal perception and sensory processing, and is involved in fear processing and the production of fear memory (Shang et al., 2019). PCG is also abnormal when comparing PD with GAD and MDD (Maggioni et al., 2019). Changes in PCG function are commonly reported in memory impairment and aging-related diseases (Kropf et al., 2019; Zeng et al., 2019). Memory is also one of the indicators for assessing aging. Anxiety and reduced social interaction can be partly attributed to memory impairment (Veluri, 2019). Memory decline will inevitably affect social communication and social behavior (Petkus et al., 2017), and one study confirms that spatial working memory is related to social factors scores (Fleck et al., 2019). Our findings indicate that disrupting the expression of TERT in mice can influence the acquisition of recent fear memory. We can boldly speculate that the pathological changes of TERT gene may affect memory acquisition and lead to social function impairment. TERT^–/–^ mice had terrible spatial memory and the TERT gene had the ability to regulate the formation of spatial memory by adjusting the number and morphological complexity of neurons (Zhou et al., 2017). TERT knockout mice generally have shorter telomeres which are typically associated with poor memory include episodic and spatial (Czepielewski et al., 2018; Powell et al., 2018), and lead to episodic memory-related learning activities defects (Valdes et al., 2010). As a result, knockout mice may have a weaker perceptive ability in the contextual conditioning chamber or impaired memory, which may lead to a reduced ability to acquire recent fear memory as quickly as the control group. Some studies have reported that poor spatial memory in mental patients is associated with reduced activation (Eckfeld et al., 2017) and reduced resting-state functional connectivity of PCG and IPL (Ren et al., 2019). Further investigation is needed to confirm the relationship between IPL/PCG, TERT, and fear memory and between memory mediated by the TERT gene and social function to refine the gene-related pathophysiological mechanism in PD aging.

Finally, some limitations to the present study should be mentioned. First, the sample size of our study was not large enough. Second, whether the methylation of hTERT in peripheral blood can directly affect brain function and whether it is consistent with gene methylation in the brain is unclear. Third, the evaluation of social function needs to be improved. In future studies, it might be helpful to include SAD as a control group to determine whether the gene methylation has a wide influence on social function.

In conclusion, our results suggest that the hTERT gene may play an important role in the pathogeny of PD aging. Memory impairment and social dysfunction are both signs of aging. This study’s findings provide evidence that the deletion of the hTERT gene attenuates the acquisition of fear memory, and hTERT methylation modulates the local brain function of postcentral gyrus to affect the social function of PD patients. These findings could potentially help us develop novel ways to predict and evaluate the aging risk of panic disorder and enrich the research field of telomere genetic imaging in PD.

## Data availability statement

The original contributions presented in this study are publicly available. This data can be found here: https://www.ncbi.nlm.nih.gov/, assesion: PRJNA793355. The data and code that support the findings of the present study are available from the corresponding author through reasonable request. The data and code sharing adopted by the authors comply with the requirements of the funding institute and with institutional ethics approval (ClinicalTrails.gov, ID: NCT03199625, URL: https://clinicaltrials.gov/).

## Ethics statement

The studies involving human participants were reviewed and approved by the Ethics Committee of the Nanjing Brain Hospital, affiliates of Nanjing Medical University. The patients/participants provided their written informed consent to participate in this study. The animal study was reviewed and approved by Institutional Animal Care and Use Committee of the Cleveland Clinic and Nanjing Medical University.

## Author contributions

CW and QZ designed the study and supervised the conduct of the study. GL, HW, and SY contributed to the data collection. HX, YW, and YZ provided methodological advice. HD and HX performed the data analysis and results interpretation. HD, CW, and QZ drafted the manuscript. All authors reviewed and approved for publication.

## References

[B1] Ain Q. Schmeer C. Penndorf D. Fischer M. Bondeva T. Forster M. (2018). Cell cycle-dependent and -independent telomere shortening accompanies murine brain aging. *Aging (Albany NY)* 10 3397–3420. 10.18632/aging.101655 30472697 PMC6286833

[B2] Alisch R. S. Van Hulle C. Chopra P. Bhattacharyya A. Zhang S. C. Davidson R. J. (2017). A multi-dimensional characterization of anxiety in monozygotic twin pairs reveals susceptibility loci in humans. *Transl. Psychiatry* 7:1282. 10.1038/s41398-017-0047-9 29225348 PMC5802687

[B3] Bartlett A. A. Singh R. Hunter R. G. (2017). Anxiety and epigenetics. *Adv. Exp. Med. Biol.* 978 145–166. 28523545 10.1007/978-3-319-53889-1_8

[B4] Cardillo G. M. De-Paula V. J. R. Ikenaga E. H. Costa L. R. Catanozi S. Schaeffer E. L. (2018). Chronic lithium treatment increases telomere length in parietal cortex and hippocampus of triple-transgenic alzheimer’s disease mice. *J. Alzheimers Dis.* 63 93–101. 10.3233/JAD-170838 29614649

[B5] Conty L. Dezecache G. Hugueville L. Grezes J. (2012). Early binding of gaze, gesture, and emotion: neural time course and correlates. *J. Neurosci.* 32 4531–4539. 10.1523/JNEUROSCI.5636-11.2012 22457500 PMC6622060

[B6] Czepielewski L. S. Massuda R. Panizzutti B. Grun L. K. Barbe-Tuana F. M. Teixeira A. L. (2018). Telomere length and CCL11 levels are associated with gray matter volume and episodic memory performance in schizophrenia: evidence of pathological accelerated aging. *Schizophr. Bull.* 44 158–167. 10.1093/schbul/sbx015 28338779 PMC5767949

[B7] de Wilde J. Kooter J. M. Overmeer R. M. Claassen-Kramer D. Meijer C. J. Snijders P. J. (2010). hTERT promoter activity and CpG methylation in HPV-induced carcinogenesis. *BMC Cancer* 10:271. 10.1186/1471-2407-10-271 20534141 PMC2904279

[B8] Eckfeld A. Karlsgodt K. H. Haut K. M. Bachman P. Jalbrzikowski M. Zinberg J. (2017). Disrupted working memory circuitry in adolescent psychosis. *Front. Hum. Neurosci.* 11:394. 10.3389/fnhum.2017.00394 28848413 PMC5550407

[B9] Emeny R. T. Baumert J. Zannas A. S. Kunze S. Wahl S. Iurato S. (2018). Anxiety associated increased CpG methylation in the promoter of Asb1: a translational approach evidenced by epidemiological and clinical studies and a murine model. *Neuropsychopharmacology* 43 342–353. 10.1038/npp.2017.102 28540928 PMC5729551

[B10] Fleck J. I. Arnold M. Dykstra B. Casario K. Douglas E. Morris O. (2019). Distinct functional connectivity patterns are associated with social and cognitive lifestyle factors: pathways to cognitive reserve. *Front. Aging Neurosci.* 11:310. 10.3389/fnagi.2019.00310 31798441 PMC6863775

[B11] Gechter J. Liebscher C. Geiger M. J. Wittmann A. Schlagenhauf F. Lueken U. (2019). Association of NPSR1 gene variation and neural activity in patients with panic disorder and agoraphobia and healthy controls. *Neuroimage Clin.* 24:102029. 10.1016/j.nicl.2019.102029 31734525 PMC6854061

[B12] Gonzalez-Giraldo Y. Forero D. A. Echeverria V. Gonzalez J. Avila-Rodriguez M. Garcia-Segura L. M. (2016). Neuroprotective effects of the catalytic subunit of telomerase: a potential therapeutic target in the central nervous system. *Ageing Res. Rev.* 28 37–45. 10.1016/j.arr.2016.04.004 27095058

[B13] Gottschalk M. G. Richter J. Ziegler C. Schiele M. A. Mann J. Geiger M. J. (2019). Orexin in the anxiety spectrum: association of a HCRTR1 polymorphism with panic disorder/agoraphobia. CBT treatment response and fear-related intermediate phenotypes. *Transl. Psychiatry* 9:75. 10.1038/s41398-019-0415-8 PMC6361931 30718541

[B14] Guilleret I. Benhattar J. (2003). Demethylation of the human telomerase catalytic subunit (hTERT) gene promoter reduced hTERT expression and telomerase activity and shortened telomeres. *Exp. Cell Res.* 289 326–334. 14499633 10.1016/s0014-4827(03)00281-7

[B15] Husain M. Nachev P. (2007). Space and the parietal cortex. *Trends Cogn. Sci.* 11 30–36. 17134935 10.1016/j.tics.2006.10.011 PMC2323620

[B16] Inoue A. Akiyoshi J. Muronaga M. Masuda K. Aizawa S. Hirakawa H. (2015). Association of TMEM132D, COMT, and GABRA6 genotypes with cingulate, frontal cortex and hippocampal emotional processing in panic and major depressive disorder. *Int. J. Psychiatry Clin. Pract.* 19 192–200. 10.3109/13651501.2015.1043133 25974322

[B17] Irle E. Barke A. Lange C. Ruhleder M. (2014). Parietal abnormalities are related to avoidance in social anxiety disorder: a study using voxel-based morphometry and manual volumetry. *Psychiatry Res.* 224 175–183. 10.1016/j.pscychresns.2014.08.013 25240316

[B18] Iurato S. Carrillo-Roa T. Arloth J. Czamara D. Diener-Holzl L. Lange J. (2017). DNA methylation signatures in panic disorder. *Transl. Psychiatry* 7:1287. 29249830 10.1038/s41398-017-0026-1 PMC5802688

[B19] Kessler R. C. Chiu W. T. Jin R. Ruscio A. M. Shear K. Walters E. E. (2006). The epidemiology of panic attacks, panic disorder, and agoraphobia in the national comorbidity survey replication. *Arch. Gen. Psychiatry* 63 415–424. 16585471 10.1001/archpsyc.63.4.415 PMC1958997

[B20] Kim K. C. Cho K. S. Yang S. M. Gonzales E. L. Valencia S. Eun P. H. (2017). Sex differences in autism-like behavioral phenotypes and postsynaptic receptors expression in the prefrontal cortex of TERT transgenic mice. *Biomol. Ther. (Seoul)* 25 374–382. 10.4062/biomolther.2016.242 28208013 PMC5499615

[B21] Kim K. C. Rhee J. Park J. E. Lee D. K. Choi C. S. Kim J. W. (2016). Overexpression of telomerase reverse transcriptase induces autism-like excitatory phenotypes in mice. *Mol. Neurobiol.* 53 7312–7328. 10.1007/s12035-015-9630-3 26696493

[B22] King K. S. Kozlitina J. Rosenberg R. N. Peshock R. M. Mccoll R. W. Garcia C. K. (2014). Effect of leukocyte telomere length on total and regional brain volumes in a large population-based cohort. *JAMA Neurol.* 71 1247–1254. 10.1001/jamaneurol.2014.1926 25090243 PMC5479062

[B23] Klapper W. Shin T. Mattson M. P. (2001). Differential regulation of telomerase activity and TERT expression during brain development in mice. *J. Neurosci. Res.* 64 252–260. 11319769 10.1002/jnr.1073

[B24] Kropf E. Syan S. K. Minuzzi L. Frey B. N. (2019). From anatomy to function: the role of the somatosensory cortex in emotional regulation. *Braz. J. Psychiatry* 41 261–269. 30540029 10.1590/1516-4446-2018-0183 PMC6794131

[B25] Lai C. H. Wu Y. T. (2016). The changes in the low-frequency fluctuations of cingulate cortex and postcentral gyrus in the treatment of panic disorder: the MRI study. *World J. Biol. Psychiatry* 17 58–65. 10.3109/15622975.2015.1017604 25789962

[B26] Lee J. Jo Y. S. Sung Y. H. Hwang I. K. Kim H. Kim S. Y. (2010). Telomerase deficiency affects normal brain functions in mice. *Neurochem. Res.* 35 211–218. 10.1007/s11064-009-0044-3 19685288

[B27] Li Z. Tang J. Li H. Chen S. He Y. Liao Y. (2014). Shorter telomere length in peripheral blood leukocytes is associated with childhood autism. *Sci. Rep.* 4:7073. 10.1038/srep07073 25399515 PMC4233346

[B28] Lindqvist D. Epel E. S. Mellon S. H. Penninx B. W. Revesz D. Verhoeven J. E. (2015). Psychiatric disorders and leukocyte telomere length: underlying mechanisms linking mental illness with cellular aging. *Neurosci. Biobehav. Rev.* 55 333–364. 10.1016/j.neubiorev.2015.05.007 25999120 PMC4501875

[B29] Maggioni E. Delvecchio G. Grottaroli M. Garzitto M. Piccin S. Bonivento C. (2019). Common and different neural markers in major depression and anxiety disorders: a pilot structural magnetic resonance imaging study. *Psychiatry Res. Neuroimaging* 290 42–50. 31279954 10.1016/j.pscychresns.2019.06.006

[B30] Magnotta V. A. Johnson C. P. Follmer R. Wemmie J. A. (2014). Functional t1rho imaging in panic disorder. *Biol. Psychiatry* 75 884–891. 10.1016/j.biopsych.2013.09.008 24157339 PMC7852409

[B31] Markowitz J. S. Weissman M. M. Ouellette R. Lish J. D. Klerman G. L. (1989). Quality of life in panic disorder. *Arch. Gen. Psychiatry* 46 984–992. 2684086 10.1001/archpsyc.1989.01810110026004

[B32] Meyer-Lindenberg A. Weinberger D. R. (2006). Intermediate phenotypes and genetic mechanisms of psychiatric disorders. *Nat. Rev. Neurosci.* 7 818–827. 16988657 10.1038/nrn1993

[B33] Michalek J. E. Kepa A. Vincent J. Frissa S. Goodwin L. Hotopf M. (2017). Genetic predisposition to advanced biological ageing increases risk for childhood-onset recurrent major depressive disorder in a large UK sample. *J. Affect. Disord.* 213 207–213. 10.1016/j.jad.2017.01.017 28233563 PMC6191533

[B34] Nomikos N. N. Nikolaidis P. T. Sousa C. V. Papalois A. E. Rosemann T. Knechtle B. (2018). Exercise. telomeres, and Cancer: “the exercise-telomere hypothesis”. *Front. Physiol.* 9:1798. 10.3389/fphys.2018.01798 30618810 PMC6305363

[B35] Oh J. E. Chambwe N. Klein S. Gal J. Andrews S. Gleason G. (2013). Differential gene body methylation and reduced expression of cell adhesion and neurotransmitter receptor genes in adverse maternal environment. *Transl. Psychiatry* 3:e218. 10.1038/tp.2012.130 23340501 PMC3566713

[B36] Perna G. Iannone G. Alciati A. Caldirola D. (2016). Are anxiety disorders associated with accelerated aging? *Neural Plast.* 2016:8457612. 26881136 10.1155/2016/8457612 PMC4736204

[B37] Petkus A. J. Reynolds C. A. Wetherell J. L. Kremen W. S. Gatz M. (2017). Temporal dynamics of cognitive performance and anxiety across older adulthood. *Psychol. Aging* 32 278–292. 10.1037/pag0000164 28333502 PMC5573587

[B38] Powell T. R. De Jong S. Breen G. Lewis C. M. Dima D. (2019). Telomere length as a predictor of emotional processing in the brain. *Hum. Brain Mapp.* 40 1750–1759. 30511786 10.1002/hbm.24487 PMC6492163

[B39] Powell T. R. Dima D. Frangou S. Breen G. (2018). Telomere length and bipolar disorder. *Neuropsychopharmacology* 43 445–453. 28621334 10.1038/npp.2017.125 PMC5729555

[B40] Powell-Wiley T. M. Gebreab S. Y. Claudel S. E. Ayers C. Andrews M. R. Adu-Brimpong J. (2020). The relationship between neighborhood socioeconomic deprivation and telomere length: the 1999-2002 national health and nutrition examination survey. *SSM Popul. Health* 10:100517. 10.1016/j.ssmph.2019.100517 31872036 PMC6909179

[B41] Ren Z. Zhang Y. He H. Feng Q. Bi T. Qiu J. (2019). The different brain mechanisms of object and spatial working memory: voxel-based morphometry and resting-state functional connectivity. *Front. Hum. Neurosci.* 13:248. 10.3389/fnhum.2019.00248 31379543 PMC6659551

[B42] Rhee J. Park K. Kim K. C. Shin C. Y. Chung C. (2018). Impaired hippocampal synaptic plasticity and enhanced excitatory transmission in a novel animal model of autism spectrum disorders with telomerase reverse transcriptase overexpression. *Mol. Cells* 41 486–494. 10.14348/molcells.2018.0145 29696935 PMC5974625

[B43] Shang C. Y. Lin H. Y. Gau S. S. (2019). The norepinephrine transporter gene modulates intrinsic brain activity, visual memory, and visual attention in children with attention-deficit/hyperactivity disorder. *Mol. Psychiatry* 26 4026–4035. 10.1038/s41380-019-0545-7 31595036

[B44] Silva P. N. Gigek C. O. Leal M. F. Bertolucci P. H. De Labio R. W. Payao S. L. (2008). Promoter methylation analysis of SIRT3, SMARCA5, HTERT and CDH1 genes in aging and Alzheimer’s disease. *J. Alzheimers Dis.* 13 173–176. 10.3233/jad-2008-13207 18376059

[B45] Sobanski T. Wagner G. (2017). Functional neuroanatomy in panic disorder: status quo of the research. *World J. Psychiatry* 7 12–33. 10.5498/wjp.v7.i1.12 28401046 PMC5371170

[B46] Syal S. Hattingh C. J. Fouche J. P. Spottiswoode B. Carey P. D. Lochner C. (2012). Grey matter abnormalities in social anxiety disorder: a pilot study. *Metab. Brain Dis.* 27 299–309. 10.1007/s11011-012-9299-5 22527992

[B47] Teyssier J. R. Chauvet-Gelinier J. C. Ragot S. Bonin B. (2012). Up-regulation of leucocytes genes implicated in telomere dysfunction and cellular senescence correlates with depression and anxiety severity scores. *PLoS One* 7:e49677. 10.1371/journal.pone.0049677 23185405 PMC3504145

[B48] Valdes A. M. Deary I. J. Gardner J. Kimura M. Lu X. Spector T. D. (2010). Leukocyte telomere length is associated with cognitive performance in healthy women. *Neurobiol. Aging* 31 986–992. 18718693 10.1016/j.neurobiolaging.2008.07.012 PMC2876308

[B49] Veluri N. (2019). A case of cognitive decline resulting from aging, temporal lobe epilepsy, and environmental factors. *Case Rep. Psychiatry* 2019:9385031. 10.1155/2019/9385031 31886001 PMC6925934

[B50] Verhoeven J. E. Revesz D. Van Oppen P. Epel E. S. Wolkowitz O. M. Penninx B. W. (2015). Anxiety disorders and accelerated cellular ageing. *Br. J. Psychiatry* 206 371–378. 25657360 10.1192/bjp.bp.114.151027

[B51] Wheater E. N. W. Stoye D. Q. Cox S. R. Wardlaw J. M. Drake A. J. Bastin M. E. (2020). DNA methylation and brain structure and function across the life course: a systematic review. *Neurosci. Biobehav. Rev.* 113 133–156. 10.1016/j.neubiorev.2020.03.007 32151655 PMC7237884

[B52] Yochim B. P. Mueller A. E. Segal D. L. (2013). Late life anxiety is associated with decreased memory and executive functioning in community dwelling older adults. *J. Anxiety Disord.* 27 567–575. 10.1016/j.janxdis.2012.10.010 23298889

[B53] Zeng Q. Luo X. Li K. Wang S. Zhang R. Hong H. (2019). Distinct spontaneous brain activity patterns in different biologically-defined alzheimer’s disease cognitive stage: a preliminary study. *Front. Aging Neurosci.* 11:350. 10.3389/fnagi.2019.00350 32009939 PMC6980867

[B54] Zhou Q. G. Liu M. Y. Lee H. W. Ishikawa F. Devkota S. Shen X. R. (2017). Hippocampal TERT regulates spatial memory formation through modulation of neural development. *Stem Cell Rep.* 9 543–556. 10.1016/j.stemcr.2017.06.014 28757168 PMC5550029

[B55] Ziegler C. Dannlowski U. Brauer D. Stevens S. Laeger I. Wittmann H. (2015). Oxytocin receptor gene methylation: converging multilevel evidence for a role in social anxiety. *Neuropsychopharmacology* 40 1528–1538. 10.1038/npp.2015.2 25563749 PMC4397412

