# Supplementary material for: Panic disorder aging characteristics: The role of telomerase reverse transcriptase gene and brain function
Source: Front Aging Neurosci. 2022 Aug 5;14:835963. doi: 10.3389/fnagi.2022.835963 (PMC9389410; doi:10.3389/fnagi.2022.835963)
Supplement: Supplementary file 1 [file Table_1.docx]

| Target | Chr | Gene | TSS | Start | End | Length | Distance | Primer sequences | |
| --- | --- | --- | --- | --- | --- | --- | --- | --- | --- |
|  |  |  |  |  |  |  |  | Fforward | Rreverse |
| CpG_1 | 5 | hTERT | 1295162 | 1294219 | 1294474 | 256 | 688 | TTGTTYGGGTGGGTTTAGGATT | ACCAACTCRACRCTACCACTCAAAC |
| CpG_2 | 5 | hTERT | 1295162 | 1294404 | 1294641 | 238 | 521 | GGTTATYGTGTTGGGTAGGTAGTTG | CTCCTTCCRCCAAATAAACCTC |
| CpG_3 | 5 | hTERT | 1295162 | 1294617 | 1294898 | 282 | 264 | GAGGTTTATTTGGYGGAAGGAG | TACRCTCCCTACTACRCAACCACTACC |
| CpG_4 | 5 | hTERT | 1295162 | 1294877 | 1295076 | 200 | 86 | GGTAGTGGTTGYGTAGTAGGGAGYGTA | TACCCCTTCACCTTCCAACTC |
| CpG_5 | 5 | hTERT | 1295162 | 1295050 | 1295309 | 260 | -147 | GGTAGTGGTTGYGTAGTAGGGAGYGTA | TACCCCTTCACCTTCCAACTC |
| CpG_6 | 5 | hTERT | 1295162 | 1295289 | 1295576 | 288 | -414 | GAGTTGGAAGGTGAAGGGGTA | CCAAAACCTCCACATCATAACC |
| CpG_7 | 5 | hTERT | 1295162 | 1295602 | 1295858 | 257 | -696 | GGTYGTTGGTTTGATTYGGAGAT | CCTCRCCACCTAAAAACCTACAA |

**Supplementary Table 1. The position of the eight CpG regions and primer sequences**

hTERT:human telomerase reverse transcriptase, Chr:Chromosome, TSS: transcription start site, Distance 2TSS:CpG site relative distance (in bp) to TSS, F forward primer, R reverse.
